# Genome-wide analysis indicates association between heterozygote advantage and healthy aging in humans

**DOI:** 10.1186/s12863-019-0758-4

**Published:** 2019-07-02

**Authors:** Ke Xu, Roman Kosoy, Khader Shameer, Sudhir Kumar, Li Liu, Ben Readhead, Gillian M. Belbin, Hao-Chih Lee, Rong Chen, Joel T. Dudley

**Affiliations:** 1 Department of Genetics and Genomic Sciences, Icahn School of Medicine at Mount Sinai, New York, NY USA; 2 Icahn Institute for Genomics and Multiscale Biology, Icahn School of Medicine at Mount Sinai, New York, NY USA; 3 Institute for Next Generation Healthcare, Icahn School of Medicine at Mount Sinai, New York, NY USA; 4 Institute for Genomics and Evolutionary Medicine, Temple University, Philadelphia, PA USA; 5 Department of Biology, Temple University, Philadelphia, PA USA; 6 Center for Excellence in Genome Medicine and Research, King Abdulaziz University, Jeddah, Saudi Arabia; 7 Department of Biomedical Informatics, Arizona State University, Tempe, AZ USA; 8 The Charles Bronfman Institute for Personalized Medicine, Icahn School of Medicine at Mount Sinai, New York, NY USA; 9 Present Address: Center for Applied Bioinformatics, St. Jude Children’s Research Hospital, Memphis, TN USA; 10 Present Address: Advanced Analytics Center, AstraZeneca, Gaithersburg, MD USA; 11 Present Address: ASU-Banner Neurodegenerative Disease Research Center, Arizona State University, Tempe, AZ USA

**Keywords:** Heterozygote advantage, Balancing selection, Fitness, Healthy aging, Human diseases, Electronic health record

## Abstract

**Background:**

Genetic diversity is known to confer survival advantage in many species across the tree of life. Here, we hypothesize that such pattern applies to humans as well and could be a result of higher fitness in individuals with higher genomic heterozygosity.

**Results:**

We use healthy aging as a proxy for better health and fitness, and observe greater heterozygosity in healthy-aged individuals. Specifically, we find that only common genetic variants show significantly higher excess of heterozygosity in the healthy-aged cohort. Lack of difference in heterozygosity for low-frequency variants or disease-associated variants excludes the possibility of compensation for deleterious recessive alleles as a mechanism. In addition, coding SNPs with the highest excess of heterozygosity in the healthy-aged cohort are enriched in genes involved in extracellular matrix and glycoproteins, a group of genes known to be under long-term balancing selection. We also find that individual heterozygosity rate is a significant predictor of electronic health record (EHR)-based estimates of 10-year survival probability in men but not in women, accounting for several factors including age and ethnicity.

**Conclusions:**

Our results demonstrate that the genomic heterozygosity is associated with human healthspan, and that the relationship between higher heterozygosity and healthy aging could be explained by heterozygote advantage. Further characterization of this relationship will have important implications in aging-associated disease risk prediction.

**Electronic supplementary material:**

The online version of this article (10.1186/s12863-019-0758-4) contains supplementary material, which is available to authorized users.

## Background

Genetic diversity within a population, often characterized by heterozygosity, is known to play an important role in conferring benefit for survival and reproduction [1]. Advantage of heterozygotes over homozygotes has been observed in many species ranging from plants to mammals [2–4]. For example, inbred lines of maize have lower heterozygosity and lower agricultural yield than their ancestors, but a cross of two different inbred lines can match or even exceed the yield of their ancestors [5]. In birds, blue tit females were demonstrated to preferentially mate with genetically dissimilar males to increase their offspring’s heterozygosity and fitness [6]. In mammals, soy sheep with lower heterozygosity are more susceptible to parasite infection and exhibit lower fitness [7].

In humans, high genetic diversity of Major Histocompatibility Complex (MHC) region conveys robust pathogen resistance on the population level and, therefore, important for fighting against infectious diseases [8, 9]. However, the role of heterozygosity is less well studied in non-MHC regions, though interesting trends are emerging. People with higher heterozygosity are reported to exhibit better health-associated traits, such as lower blood pressure and lower LDL cholesterol level compared to people with lower heterozygosity [10]. There have also been reports of significant association between genome-wide heterozygosity and risk of death based on large number of genetic markers and samples [11].

While it is largely accepted that inbreeding reduces heterozygosity and fitness (inbreeding depression) and outbreeding does the opposite (heterosis), the genetic mechanism underlying the heterozygosity-fitness correlation (HFC) is still under debate, and poorly studied in humans [12]. There are two major competing proposed mechanisms to explain the observed HFC. One mechanism suggests that heterozygous state of a locus has better survival advantage than either homozygous state (heterozygote advantage, or overdominance), such as the well-known example of the protective effect against malaria in the sickle-cell allele carriers [13, 14]. The other mechanism suggests that it is mainly due to reduced chances of deleterious recessive alleles to be found in homozygous states in outbred individuals.

We set out to investigate HFC and its mechanism in human non-MHC loci by using two genetically matched cohorts: a Wellderly cohort representing a healthy-aged population and the Mount Sinai Bio*Me* Biobank cohort representing a general population. Such datasets have only become available recently because of a growing interest in understanding the genetic basis of wellness, or health, as opposed to the disease-centered genome-wide association studies (GWAS) [15–17]. The Wellderly cohort consists of people who are over 80 years old with no history of chronic diseases or taking chronic medications [18]. As described in the publication on the Wellderly study, healthy aging is distinct from exceptional longevity. Therefore, Wellderly can be viewed as a cohort with better health and greater fitness compared to general population. In our case, Mount Sinai Bio*Me* Biobank cohort represents a US-based general outpatient population [19], which is a mixture of ill and healthy people.

We found that higher heterozygosity is associated with better human health, and the association is more likely to be explained by heterozygote advantage than by compensation for deleterious recessive alleles.

## Results

### Similar allele frequency but distinct heterozygosity between genetically matched Wellderly and biobank individuals

Before doing any genetic comparisons between the Wellderly and the Biobank cohorts, we first determined their population structure using 1000 Genomes Project’s European populations as reference [20], and all of the following analyses were restricted to individuals of non-Ashkenazi Jewish European ancestry. Principal component analysis (PCA) revealed that while majority of the Wellderly individuals overlapped with the CEU (Utah residents with Northern and Western ancestry) and GBR (British in England and Scotland) populations, Biobank individuals displayed higher diversity (Additional file 1: Figure S1), likely reflecting the distinct demographic of New York City. In order to remove the influence of underlying population structure when comparing Wellderly and Biobank cohorts, we genetically matched the two cohorts following Gregerson et al. (see Materials and methods for details) [21]. After the 1:1 matching, 426 pairs of individuals were retained from the original 454 Wellderly individuals and 1107 Biobank individuals (Additional file 2: Figure S2). To test if the genetic matching is effective, we computed genomic inflation factor (λ_gc_) before and after the matching (1.3 and 1.01, correspondingly), suggesting the effective removal of the systematic bias introduced by population structure. After filtering, 228,606 noncoding SNPs passed the stringent quality control (QC), and the minor allele frequencies (MAF) were highly similar between the two cohorts (Additional file 3: Figure S3A), suggesting no systematic bias potentially introduced by difference in genotyping methods.

**Fig. 1:**
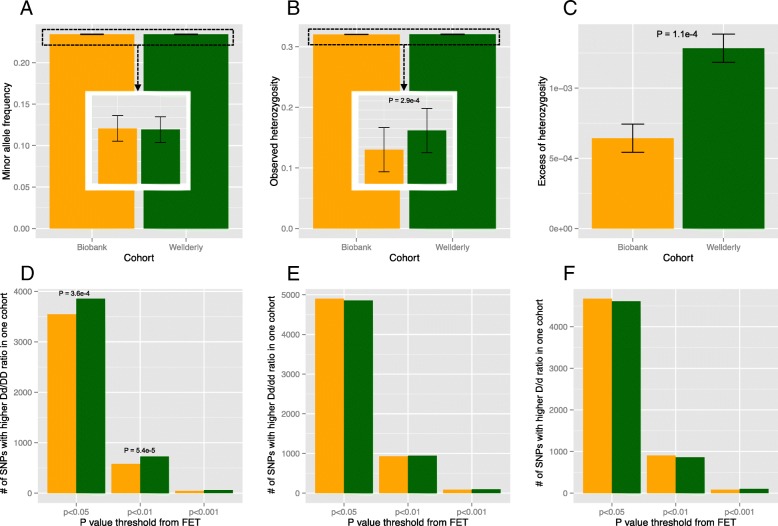
Comparisons of noncoding SNPs between Biobank (orange) and Wellderly (green). **a** Mean minor allele frequency. **b** Mean observed heterozygosity. **c** Mean excess of heterozygosity. **d** Number of SNPs showing higher ratio of Dd/DD (D: minor allele; d: major allele) in Biobank or Wellderly under different nominal *P* value cutoffs from Fisher’s Exact Test (FET). **e** Number of SNPs showing higher ratio of Dd/dd in Biobank or Wellderly under different nominal *P* value cutoffs from FET. **f** Number of SNPs showing higher ratio of D/d in Biobank (Orange) or Wellderly (Green) under different nominal *P* value cutoffs from FET. The error bars represent standard errors. *P* values shown are raw values but with FDR < 0.05

To compare the heterozygosity between the two cohorts, we focused on noncoding SNPs. For each SNP in each cohort, we calculated the following: observed heterozygosity (HET_O_), expected heterozygosity (HET_E_), and excess of heterozygosity computed as (HET_O_ - HET_E_)/HET_E_. Positive excess of heterozygosity would indicate that HET_O_ is higher than HET_E_ in that cohort. As expected, HET_E_ were highly similar between the two cohorts because HET_E_ is determined by MAF (Additional file 3: Figure S3B). Similarly, HET_O_ were also highly correlated between the two cohorts because HET_O_ is also largely driven by MAF (Additional file 3: Figure S3C). In contrast, the excess of heterozygosity was not correlated at all between the two cohorts (Additional file 3: Figure S3D), demonstrating its independence from MAF. To test if MAF, HET_O_, and excess of heterozygosity statistically differ between the two cohorts, we applied paired Mann-Whitney U test to each measure. As expected, there was no significant difference in MAF between the two cohorts (Fig. 1a, *P* = 0.338). HET_O_, however, was significantly higher in Wellderly than in Biobank (Fig. 1b, *P* = 0.0003) despite being highly dependent on MAF. And excess of heterozygosity comparison revealed even larger difference between the two cohorts, with Wellderly being almost twice higher than Biobank (Fig. 1c, *P* = 0.0001). Therefore, these results support our hypothesis that healthy-aged individuals harbor greater genomic heterozygosity than the general population.

Although the paired Mann-Whitney U test results demonstrated that overall distribution of heterozygosity is significantly higher in the Wellderly, they did not provide SNP level significance. In order to compare the heterozygosity difference for each SNP, we conducted genotype association tests. Specifically, for a SNP with ‘D’ being minor allele and ‘d’ being major allele, we compared number of heterozygous genotype ‘Dd’ and number of homozygous genotype ‘DD’ between the two cohorts using Fisher’s exact test (FET). If heterozygosity were similar between the two cohorts, we would expect to find similar number of SNPs having higher Dd/DD (# heterozygotes vs. # minor allele homozygotes) ratio in Biobank or in Wellderly. Instead, under the threshold of nominal *P* < 0.05 of FET, we found significantly higher number in Wellderly than in Biobank (3855 vs. 3547, *P* = 3.6e-04, binomial test, Fig. 1d). Similarly, under the threshold of nominal *P* < 0.01 of FET, we also found significantly higher number in Wellderly (728 vs. 581 in Biobank, *P* = 5.4e-05, binomial test, Fig. 1d). Under the threshold of nominal *P* < 0.001 of FET, we found no significant difference between the two cohorts due to greatly reduced sample sizes (60 in Wellderly vs 45 in Biobank, Fig. 1d). As a comparison, we also compared the ratio of Dd/dd (# heterozygotes vs. # major allele homozygotes) between the two cohorts, and we found no significant difference of the number of significant SNPs between the two cohorts under any nominal *P* value thresholds of FET (Fig. 1e). In addition, we found no significant difference between the two cohorts for the number of SNPs with nominal significance from allelic association tests (D/d) at any significance thresholds (Fig. 1f), which again indicates no allelic difference between the two cohorts. Addition of principal components to control for population structure to allelic association tests via logistic regression yielded similar results.

In order to discount the possibility that the differences between the two cohorts may be due to a small number of loci, we accounted for the effect of linkage disequilibrium (LD) between the tested markers by repeating the above analyses using LD pruned SNPs (*r*^2^ < 0.5), retaining 147,533 SNPs. We found largely consistent patterns with the above results, but the estimates of significance were lower, possibly due to reduced sample size. For example, the excess of heterozygosity was still significantly higher in Wellderly than in Biobank (*P* = 0.019, paired Mann-Whitney U test, Additional file 4: Figure S4A). And Wellderly still had significantly larger number of SNPs with higher Dd/DD ratio under nominal *P* < 0.01 of FET (458 vs 362, *P* = 9.0e-04, binomial test, Additional file 4: Figure S4B). These results suggest that the heterozygosity difference is genome-wide and is not limited to a few genomic regions with high LD.

### Evidence for heterozygote advantage

**Fig. 2:**
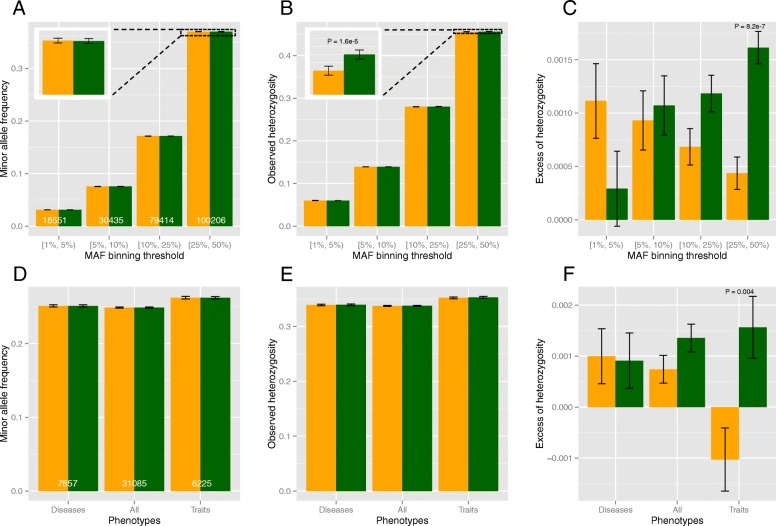
Stratified comparisons of noncoding SNPs between Biobank (orange) and Wellderly (green) for minor allele frequency (MAF) (**a** and **d**), observed heterozygosity (**b** and **e**), and excess of heterozygosity (**c** and **f**). All the bars and error bars represent sample means and their standard errors. The stratification is based on combined MAF (**a**, **b**, **c**) and associated phenotypes (**d**, **e**, **f**) separately. Numbers at the bottom of bars of panel A are the SNP numbers in different MAF bins. Numbers at the bottom of bars of panel D are the numbers of SNPs associated with selected complex diseases (Diseases), selected complex traits (Traits), and all the complex diseases and traits combined (All). *P* values shown are raw values but with FDR < 0.05

The higher heterozygosity in Wellderly can be explained by two mechanisms: 1) compensation for deleterious recessive alleles; and 2) heterozygote advantage, or overdominance. While it is difficult to directly test for the second mechanism, it is possible to examine the first one. If the first mechanism is true, we should observe greater heterozygosity difference for the low-frequency alleles because they are more likely to be under purifying selection than common alleles [12, 22]. To examine this, we binned the SNPs into four categories based on their combined MAF: 0.01 ≤ MAF < 0.05, 0.05 ≤ MAF < 0.1, 0.1 ≤ MAF < 0.25, and 0.25 ≤ MAF < 0.5. For each bin, we compared MAF, HET_O_, and excess of heterozygosity between the two cohorts. We found that for the first three bins, MAF and HET_O_ were all extremely similar between the two cohorts (Fig. 2a and b). For the fourth bin, however, while MAF was still similar, HET_O_ was significantly higher in Wellderly (*P* = 1.588e-05, paired Mann-Whitney U test), and excess of heterozygosity was almost four times higher in Wellderly than in Biobank (*P* = 8.236e-07, paired Mann-Whitney U test, Fig. 2c). Interestingly, in the first bin, with the lowest allele frequency, Wellderly actually exhibits lower excess of heterozygosity compared to Biobank (*P* = 0.04, paired Mann-Whitney U test, Fig. 2c). These results are therefore opposite from the pattern predicted by the first mechanism.

We next examine whether GWAS-identified genetic variants show heterozyogosity difference between the two cohorts. To do this, we downloaded all the SNPs with nominal *P* < 1e-3 in their GWA studies from GWASdb [23, 24], among which 31,085 SNPs were found in our data set. We found no significant difference on MAF, HET_O_, and excess of heterozygosity between the two cohorts (Fig. 2 d, e, and f). However, since GWAS phenotypes include both complex diseases and complex traits such as BMI and height, it is possible that signal from one category is masked by the other. To mediate this, we extracted 7857 SNPs associated with a set of complex diseases and 6225 SNPs associated with a set of phenotypic traits (see Materials and methods for details). Interestingly, we find that the excess of heterozygosity is significantly higher in Wellderly for the SNPs associated with complex traits but not for the SNPs associated with complex diseases (Fig. 2f). Since most GWAS hits are identified using additive model only [25, 26], our results suggest that intermediate levels of complex traits through heterozygous state convey advantages to human health.

We also repeated the above two analyses using the LD pruned SNPs, and the resulting patterns of excess of heterozygosity are consistent with the above (Additional file 4: Figure S4C and D).

### Correlation between individual heterozygosity rate and 10-year survival probability

**Fig. 3:**
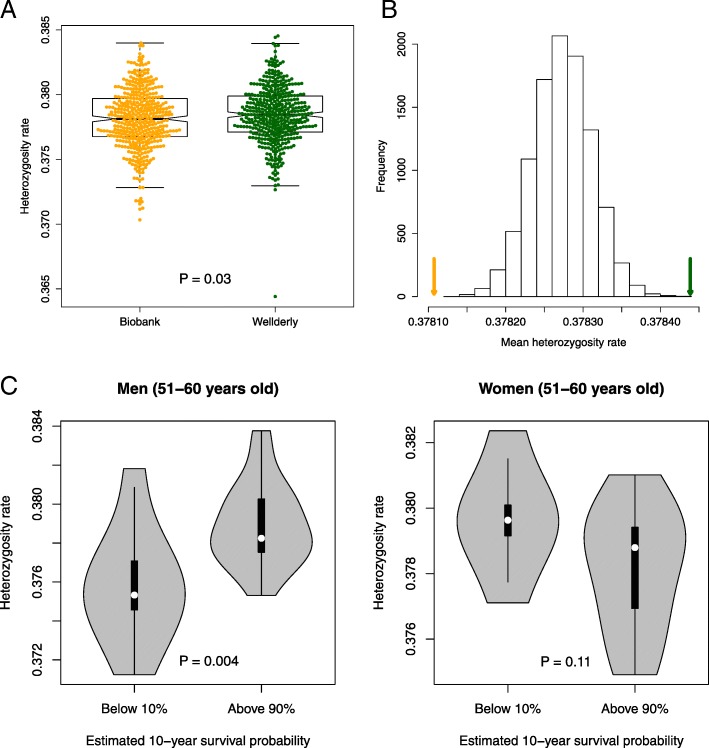
Individual heterozygosity rate. **a** Individual heterozygosity rates of Biobank (orange) and Wellderly (green). **b** Average individual heterozygosity rate between Biobank (orange arrow), Wellderly (green arrow), and 10,000 permutations. **c** Comparison of individual heterozygosity rate between group of individuals (51–60 years old) with < 10% estimated 10-year survival probability and group of individuals (51–60 years old) with > 90% estimated 10-year survival probability in men and women of the Biobank cohort, separately. We restrict the comparison to the 51–60 year olds because no individual older than 60 years has estimated 10-year survival probability > 90% in the Biobank cohort

In addition to analyzing the difference in heterozygosity on SNP level, we could also analyze it on the level of an individual person. Specifically, we could calculate individual heterozygosity rate as the proportion of heterozygous sites out of all examined sites. As most of the heterozygosity difference was observed for common markers, we used SNPs with combined MAF > 0.1 (179,622 SNPs included) to calculate individual heterozygosity rate. We found that Wellderly showed significantly higher heterozygosity rate than Biobank (*P* = 0.03, Mann-Whitney U test, Fig. 3a). To further examine the statistical significance of the differences in the individual heterozygosity rate between the two cohorts, we did permutation analysis by randomly choosing 426 genotypes from the combined 852 genotypes for each SNP, and calculating individual heterozygosity rates for the newly generated individuals, repeated 10,000 times. We compared the mean heterozygosity rate of Biobank individuals and that of Wellderly individuals with those from the permutations, and found that the mean heterozygosity rate of Biobank was significantly smaller than those from permutations (*P* < 1e-4, Fig. 3b) and heterozygosity rate of Wellderly was significantly larger than those from permutations (*P* < 1e-4, Fig. 3b).

With electronic health record (EHR) data available for the Biobank cohort, we were able to test whether individuals’ heterozygosity rates are associated with their health conditions. Although there is no gold standard to quantify how healthy a person is, we could utilize a number of existing scoring schemes to characterize how sick a patient may be [27–29]. One such score is Charlson probability [30, 31], which estimates patient’s 10-year survival probability based on their age and comorbidity (See Materials and methods for details). We therefore computed Charlson probability for the 359 individuals remaining after QC (187 males and 172 females, aged between 51 to 80 years), and tested whether individual heterozygosity rate is significantly associated with Charlson probability. Specifically, in the utilized multiple linear regression model the response variable was Charlson probability, and predictors included heterozygosity rate, age, comorbidity score, gender, and five principal components accounting for the population structure. As expected, age and comorbidity score were the two most significant predictors. We found that individual heterozygosity rate indeed had a positive effect on Charlson probability, but the significance was marginal (*P* = 0.06, Table 1). Because men and women have different morbidity and mortality, we then the two genders separately. Interestingly, we found that heterozygosity rate was a statistically significant predictor in men but not in women (*P* = 0.01 and 0.7 separately, Table 1). Specifically, we found that individuals with above 90% Charlson probability have significantly higher heterozygosity rate than those with below 10% Charlson probability in men (*P* = 0.004, Mann-Whitney U test, Fig. 3c), but there is no such significant difference in women (*P* = 0.11, Mann-Whitney U test, Fig. 3c). Note that this comparison is performed on individuals in a same age group: 51–60 years. We did not perform the same analysis in other age groups because no individual older than 60 years has estimated Charlson probability greater than 90%.

**Table 1:** Coefficient (and *P* value) of each predictor in the multiple linear regression model of 10-year survival probability of Biobank individuals

	All individuals (359)	Males only (187)	Females only (172)
Heterozygosity rate	11.92 (0.06)	21.78 (0.01)	3.50 (0.70)
Age	−0.02 (1.06e-16)	−0.02 (8.93e-10)	− 0.02 (3.34e-08)
Disease score	−0.03 (1.02e-28)	− 0.03 (9.25e-22)	−0.02 (2.92e-11)
Gender	−0.03 (0.35)	NA	NA
PC1	0.002 (0.28)	0.01 (0.04)	−0.0005 (0.86)
PC2	−0.001 (0.57)	−0.001 (0.80)	− 0.002 (0.45)
PC3	−0.002 (0.26)	− 0.003 (0.23)	−0.002 (0.57)
PC4	−0.001 (0.58)	0.003 (0.21)	−0.005 (0.11)
PC5	−0.001 (0.47)	0.001 (0.77)	−0.004 (0.21)

To confirm that the observed results are not due to a random MAF threshold used to filter the genetic markers used in the analyses, we repeated the above analyses using the SNPs with combined MAF > 0.25 (100,206 SNPs) instead of combined MAF > 0.1, and found consistent results. The Wellderly cohort still showed significantly higher heterozygosity rate than the Biobank cohort (*P* = 0.005, Mann-Whitney U test). Heterozygosity rate was still a significant predictor to Charlson probability in men but not in women in the Biobank cohort (*P* = 0.026 and 0.997 separately).

### Heterozygosity difference in coding SNPs

For the coding SNPs, we focused on nonsynonymous sites – 7697 nonsynonymous SNPs passed the same filtering criteria applied to the noncoding SNPs. Unlike the noncoding SNPs, the HET_O_ or excess of heterozygosity of these nonsynonymous SNPs was similar between the two cohorts except for HET_O_ under the bin of 0.1 ≤ MAF < 0.25 (Table 2). This could be explained by most nonsynonymous sites being under strong purifying selection, with mutations at nonsynonymous sites potentially contributing to severe Mendelian diseases. Particularly, for mutations with dominant effect on phenotypes, i.e., dominant diseases, we would not expect to see heterozygosity difference between the two cohorts. Therefore, we examined the SNPs in genes implied in autosomal recessive or autosomal dominant diseases separately. We used OMIM-collected recessive and dominant disease genes curated by Petrovski et al. for this purpose [32, 33] (see Materials and methods for details). Interestingly, we found that overall HET_O_ was significantly higher in Wellderly in recessive genes yet significantly lower in Wellderly in dominant genes (Table 2), but excess of heterozygosity showed no significant difference in either gene set. When we stratified the analyses by different MAF bins, we found that the difference in recessive disease genes was primarily observed for the high-frequency alleles (0.25 ≤ MAF < 0.5) (Table 2), yet the difference in dominant disease genes was primarily observed for the low-frequency alleles (0.01 ≤ MAF < 0.05) (Table 2), consistent with the pattern we observed in the noncoding SNPs. These results demonstrated that not only Wellderly had higher heterozygosity for SNPs under less purifying selection (high frequency SNPs in recessive disease genes), but was also depleted with highly deleterious alleles (low frequency SNPs in dominant disease genes) [34].

**Table 2:** *P* values from paired Mann-Whitney U Test between Biobank and Wellderly for different subsets of nonsynonymous SNPs

	All genes				OMIM recessive genes				OMIM dominant genes			
	SNP#	MAF	HET_O_	F^a^	SNP#	MAF	HET_O_	F^a^	SNP#	MAF	HET_O_	F^a^
0.01 ≤ MAF < 0.05	3731	0.107	0.201	0.750	219	0.053	0.056	0.648	87	**9.7e-4** ^**c**^	**0.002** ^**c**^	0.07
0.05 ≤ MAF < 0.1	1243	0.638	0.459	0.095	81	0.089	0.688	0.189	30	0.155	0.430	0.626
0.1 ≤ MAF < 0.25	1548	0.049	**0.003** ^**b**^	0.101	66	0.645	0.710	0.954	35	0.276	0.30	0.518
0.25 ≤ MAF < 0.5	1175	0.126	0.133	0.359	61	0.401	**0.007** ^**b**^	0.015^b^	20	0.896	0.856	0.588
Total	7697	0.30	0.165	0.608	427	0.612	0.042^b^	0.112	172	0.053	0.035^c^	0.133

*P* values in bold pass multiple testing correction (FDR adjusted *P* < 0.1)

^a^F: excess of heterozygosity

^b^Direction: Biobank < Wellderly

^c^Direction: Biobank > Wellderly

**Fig. 4:**
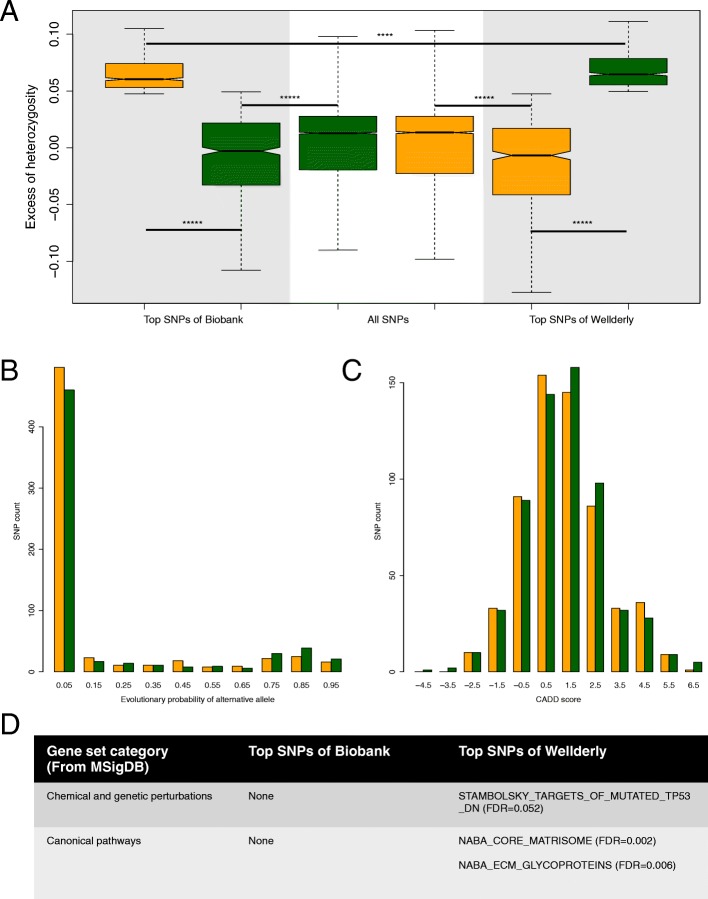
Analyses of nonsynonymous SNPs. **a** Excess of heterozygosity comparison between Biobank (orange) and Wellderly (green) for SNPs with top 10% excess of heterozygosity in Biobank (left shaded area) and SNPs with top 10% excess of heterozygosity in Wellderly (right shaded area). **** *P* < 1e-4; ***** *P* < 1e-10, Mann-Whitney U test. **b** Evolutionary probability comparison between the top SNPs in Biobank (orange) and the tops SNPs in Wellderly (green). **c** CADD score comparison between the top SNPs in Biobank (orange) and the top SNPs in Wellderly (green). **d** Significantly enriched gene sets for genes involving the top SNPs in Biobank and genes involving the top SNPs in Wellderly

Since neither observed nor excess heterozygosity was significantly different between the two cohorts for the nonsynonymous SNPs, we next investigated SNPs with the highest excess of heterozygosity in each cohort (denoted as ‘top SNPs’ below). We focused on SNPs with the top 10% of excess of heterozygosity in each cohort – 768 and 743 top SNPs were picked in Biobank and Wellderly, separately. After removing 128 SNPs shared by the two SNP sets, the sets were reduced to 640 SNPs in 560 genes for Biobank and 615 SNPs in 549 genes for Wellderly. Interestingly, for the remaining top SNPs in each cohort, their excess of heterozygosity was not only significantly lower in the other cohort but also below the average of all the SNPs in the other cohort (Fig. 4a), suggesting that the remaining set of top SNPs are unique to each cohort. Equally interesting is that the top SNPs in Wellderly had significantly higher excess of heterozygosity than the top SNPs in Biobank (Fig. 4a).

Given the mutual exclusivity of the two sets of top SNPs, we next investigate whether they have distinct evolutionary history or pathogenicity. To compare evolutionary history we use the evolutionary approach informed scores that represent evolutionary probability (EP) of each possible allele at a given nonsynonymous position using protein sequence alignment of 46 vertebrates, independent of human polymorphism data [35]. High EP suggests that an allele is evolutionarily permissible, and thus not under strong purifying selection and not likely to be pathogenic. We compared the minor alleles EPs between all nonsynonymous SNPs, the top SNPs in Biobank, and the top SNPs in Wellderly. We found that both sets of top SNPs had significantly higher minor allele EPs than the rest of SNPs (*P* < 1e-4, Mann-Whitney U test). While the difference between the two sets of top SNPs was not significant, top SNPs in Wellderly harbored less low EPs and more high EPs compared to top SNPs in Biobank (Fig. 4b). To compare pathogenicity we use the Combined Annotation Dependent Depletion (CADD) scores [36], where alleles with high scores are deemed to be deleterious or pathogenic. We found that the top SNPs in both Biobank and Wellderly had significantly lower CADD scores than the rest of SNPs (*P* < 0.01, Mann-Whitney U test), however, there was no clear trend in the CADD score distribution between the two sets of top SNPs (Fig. 4c). A recent study shows that CADD scores have limited power to classify pathogenic alleles at a given position [37].

To examine the function of the genes with the highest excess, we applied gene set enrichment analyses to the top SNPs-involved genes in each cohort (denoted as ‘top genes’ below). We tested against multiple gene set collections from Molecular Signatures Database (MSigDB) including Hallmark, chemical and genetic perturbations, canonical pathways, GO biological process, GO cellular component, GO molecular function, and immunologic signatures [38]. Interestingly, we found that the top genes in Wellderly are significantly enriched in several gene sets (FDR < 0.1), including STAMBOLSKY_TARGETS_OF_MUTATED_TP53_DN from chemical and genetic perturbations [39], and NABA_CORE_MATRISOME and NABA_ECM_GLYCOPROTEINS from canonical pathways [40] (Fig. 4d). Notably, extracellular matrix and glycoproteins have been reported to be under long-term balancing selection [41, 42]. In comparison, the top genes in Biobank were not significantly enriched in any gene set.

## Discussion

Human longevity has been an active area of genetic research, but to our knowledge Wellderly study is the first genetic study with an emphasis on healthy aging. Lifespan is different from healthspan. Although our life expectancy has increased steadily in the past decades, it is more attributed to better medical care and social support rather than improved health [43, 44]. Also, as demonstrated in the Wellderly study, genetics of healthy aging is distinct from that of exceptional longevity [18]. Therefore, the Wellderly cohort is a cohort that well represents longer healthspan compared to general populations. While previous studies find association between increased heterozygosity rate and lower blood pressure, lower total/LDL cholesterol, and lower risk of deaths in general populations [10, 11], our study provides direct evidence that genome-wide heterozygosity is higher in healthy-aged people compared to a general population. Since our goal is to specifically compare SNP heterozygosity between the two cohorts, we focused on excess of heterozygosity instead of observed heterozygosity because the latter, but not the former, is affected by allele frequency. In fact, in almost all comparisons the excess of heterozygosity differences were consistent with the observed heterozygosity differences but with more statistically significant evidence.

An important follow-up question is whether the increased heterozygosity in Wellderly is due to benefits of being heterozygous or due to purifying selection against homozygous state of deleterious recessive alleles. To answer this question, we first divided the SNPs into different bins based on their combined MAF. We observed significantly higher heterozygosity in the Wellderly cohort only in the bin with the highest MAF. Since it is unlikely that common alleles are more deleterious than low frequency alleles [22, 45], our result suggests that the underlying mechanism of increased heterozygosity is not due to compensation for deleterious recessive alleles [12, 46]. We then divided our SNPs into complex disease-associated SNPs and complex trait-associated SNPs, and we only observed significantly higher heterozygosity in the Wellderly cohort in the complex trait-associated SNPs, which may be explained by heterozygous alleles conferring optimal, i.e., intermediate, level of vital traits such as blood pressure. In fact, one theoretical study suggests that heterozygote advantage should be common during adaptation because heterozygous state prevents the overshooting of the optimal gene expression level for those regulatory mutations with large effect [47]. A recent study based on experimental data proposes that regulatory heterozygotes can reduce extrinsic expression noise so that cell population homogeneity gets enhanced [48]. It is also possible that antagonistic pleiotropy, i.e., alleles that are beneficial for individual fitness at reproductive age may be deleterious in later life, plays an important role in the link between higher heterozyogisty and healthy aging. Several empirical examples of antagonistic pleiotropy have been shown and they suggest widespread existence such alleles in the human genome [49].

In nonsynonymous SNPs, we found that Wellderly SNPs with the highest excess of heterozygosity were enriched in genes encoding extracellular matrix (ECM), especially ECM glycoproteins. ECM is a dynamic structure that provides physical support for tissue integrity and constantly remodeled to maintain tissue homeostasis. Components of ECM are involved in several critical cellular processes and can lead to numerous human diseases including fibrosis and cancer when dysregulated [50]. It is also important to note that ECM genes are one of the few targets under balancing selection [42], and membrane glycoproteins, alongside the MHC region, were even demonstrated to be under ancient balancing selection shared between humans and chimpanzees [41]. In contrast, the genes containing SNPs with the highest excess of heterozygosity in Biobank did not present any biological enrichment. The difference in gene set enrichment between the two cohorts suggest that the heterozygosity in human population may be particularly beneficial for genes involved in distinct biological processes. And because heterozygote advantage is one of the mechanisms of balancing selection, it argues against purifying selection against homozygotes of deleterious recessive alleles being the main mechanism, consistent with the evidence shown in the noncoding SNPs analyses.

In addition to demonstrating the overall difference in heterozygosity between the two cohorts, we also investigated correlation between individual heterozygosity rate and predicted survival probability within the Biobank cohort, which was significant in males but not in females. Our survival probabilities were estimated by Charlson probabilities, representing 10-year survival expectation based on the person’s age and comorbidities and, therefore, different from those based on actual number of deaths as used in other study [11]. The gender difference observed in our study could be due to a variety of reasons such as different morbidity and mortality for many diseases between men and women. Importantly, although women have longer lifespan than men, they generally have poorer health then men, i.e., the mortality-morbidity paradox (reviewed in [51]), with one potential explanation being that men with poor health are more likely to die compared to women with the same conditions. And perhaps it is because of this reason men showed significant association between individual heterozygosity rate and predicted survival probability, while the association in women might be masked by some unknown protective mechanisms. A limitation in our study is that due to lack of phenotype data from the Wellderly cohort, lifestyle factors such as history of smoking, physical activity, and educational attainment were not controlled for in our analyses and may potentially confound our results. Future studies including such individual-level lifestyle data can help strengthen our findings.

We are aware that our results may be subject to batch effect between the two cohorts, primarily due to different platforms used to call variants. Specifically, Biobank variants were identified by genotyping arrays from Illumina and Wellderly variants were detected by whole genome sequencing by Complete Genomics. There is no effective way to completely remove the difference based on our study design, but multiple lines of evidence suggest that our results are not likely to be biased by the platform difference. First, as we showed in Fig. 1, Fig. 2, and Table 2, the examined SNPs had similar MAF between the two cohorts. Second, higher heterozygosity in Wellderly was observed primarily for the high frequency variants, while the genetic variants most susceptible to inter-platform differences are more likely to be of low frequency. In fact, some of the comparisons showed the opposite direction of heterozygosity distribution, suggesting that there is no systemic bias in allele calling between the two cohorts. Last but not least, we only utilized genetic variants without any missing calls and with combined MAF greater than 0.01, which aimed to retain only the SNPs with highest confidence in allele calling so as to minimize the potential genotyping method bias. For these reasons we believe that our results are not biased by the differences in the variant calling between the two cohorts.

## Conclusions

By using a recently sequenced healthy aging cohort as a proxy for better health and fitness in humans, we demonstrated that 1) healthy-aged individuals have significantly higher genomic heterozygosity than the general population, and that 2) individuals with higher heterozygosity rate have higher 10-year survival probability in men of similar ages. We also provided evidence that the heterozygote advantage is likely to be the driving force for the increased heterozygosity of the healthy-aged people. Understanding the relationship between genomic heterozygosity and healthspan can shed light on future research on aging and disease risk prediction.

## Methods

### Genotype data processing

Whole genome sequencing of 600 Wellderly individuals were performed by Complete Genomics and variants were called by cgatools v.2.0.1 – v.2.0.4 [18]. Stringent variant filtrations were then applied (details can be found in Experimental Procedures section in [18]). Among the 600 Wellderly individuals, we picked 454 individuals that are of greater than 95% European ancestry and a maximum relatedness of 12.5% [18]. We then removed variants that were labeled as VQLOW in any of the individuals. VCFtools were used to convert the data from VCF format to Plink format [52].

Whole genome genotyping of 11,212 Mount Sinai Bio*Me* Biobank participants were performed by Illumina OmniExpress and HumanExome BeadChip arrays. Filtering was applied on individuals based on call rate, inbreeding coefficient, gender discordance between Biobank and EHR, and other factors. SNP QC was run through zCall using z-score threshold 7 [53]. Further variant filtering removed SNPs that 1) had call rate < 95%; 2) had no minor alleles; 3) were not in Hardy-Weinberg equilibrium (HWE) (*P* < 5e-5); and 4) deviated from 1 kg (< 40% vs > 60% and vice versa). Related individuals were then removed (PI_HAT > 0.2). The final data set include 10,511 individuals and 866,864 SNPs. We determined global proportions of European ancestry, African ancestry, and Native American ancestry per individual using the ADMIXTURE algorithm with a putative ancestral population number three and five-fold cross validation [54, 55]. For individuals of European ancestry, we also determined their Ashkenazi Jewish ancestry by combining self-reported information and ADMIXTURE runs. In the end, we retained 1107 unrelated Biobank individuals that are of greater than 90% European ancestry and of non-Ashkenazi Jewish ancestry.

### Population structure

To determine the population structure of the 454 Wellderly individuals and 1107 Biobank individuals, we used the common variants shared among Biobank cohort, Wellderly cohort, and 379 individuals with European ancestry from 1000 Genomes Project Phase 1 [20]. Specifically, we extracted all autosomal SNPs with MAF > 0.2 or > 0.01 from the three cohorts, excluding the MHC region (chr6: 25,000,000 - 35,000,000), nonsynonymous SNPs, and SNPs that failed HWE test (*P* < 0.001). Since the variants from the three cohorts were independently called, we only kept the SNPs with the same alternative alleles and with no missing genotypes for all the three cohorts. Next, the markers were subject to LD-based pruning by applying a sliding window of 50 SNPs and a forward shift of five SNPs at each step retaining SNPs with *r*^2^ < 0.5 [56]. Consequently, 70,622 SNPs were retained under the MAF > 0.2 threshold and 141,892 SNPs were retained under the MAF > 0.01 threshold. PCA (implemented in R [57]) on these two sets of markers yielded similar population structures (Additional file 1: Figure S1). We therefore used the PCA results generated by the SNPs under the MAF > 0.2 threshold for the rest of the analyses.

### Genetic matching between the biobank and Wellderly cohorts

As shown by the PCA plot (Additional file 2: Figure S2A), the Biobank cohort is more diverse than the Wellderly cohort even though they are both of European ancestry. To genetically match the two cohorts, we applied a previously described method that sequentially picks the best-matched Biobank individual for each Wellderly individual utilizing PCA results [21]. Specifically, starting from a random Wellderly individual, we calculated the cumulative distance to each of the Biobank individuals by summing the eigenvalue differences for the first six principal components multiplied by the amount of variance explained by each component. The Biobank individual with the smallest cumulative distance was selected as the best match to that Wellderly individual, and the matched pair was removed from the next round of matching, resulting in 454 well matched pairs. We repeated this procedure for 10 times with a different order of Wellderly individuals each time. The 10 repetitions yielded very similar results and we adopted the one that has the lowest overall distance of all the pairs (Additional file 2: Figure S2B). We plotted the distances of all the resulting matched pairs (Additional file 2: Figure S2C), and removed the pairs with large cumulative distances > 900 as the pairs above this level did not have a particularly good match between the Wellderly and Biobank samples. This process resulted in 426 matched pairs (Additional file 2: Figure S2D), with the genomic inflation factors (λ_gc_) of 1.3 and 1.01 before and after the genetic matching, indicating that we effectively removed the effect of population stratification between the two cohorts [58, 59].

### SNP frequency, heterozygosity and individual heterozygosity rate

For the matched 426 pairs of samples, we picked the SNPs that satisfy the following criteria: 1) both cohorts share the same polymorphic sites and have the same alternative alleles; 2) there is at least one alternative allele in each cohort; 3) no missing genotypes in any cohort; 4) on the autosomes excluding MHC region; 5) in HWE (*P* > 0.001); and 6) combined MAF > 1%. In total, we obtained 228,606 non-coding SNPs and 7697 nonsynonymous SNPs. In addition, the 228,606 noncoding SNPs were LD pruned (*r*^2^ < 0.5) based on the combined genotypes of the two cohorts using Plink [56], resulting in 147,533 SNPs.

Observed and expected heterozygosity were calculated using Plink [56]. The excess of heterozygosity is defined as *F* = (HET_O_ - HET_E_)/HET_E_, where HET_O_ is the observed heterozygosity and HET_E_ is the expected heterozygosity. Individual heterozygosity rate is defined as the proportion of heterozygous sites among the non-coding SNPs with combined MAF > 0.1 (179,622 SNPs) or with combined MAF > 0.25 (100,206 SNPs). Since we focused on exactly the same group of SNPs for each individual with no missing genotypes, there was no need to standardize the heterozygosity rate. All tests comparing the values between the Biobank and Wellderly cohorts were two-sided unless otherwise specified.

### Disease- and trait-associated SNPs

SNPs associated with complex diseases and phenotypic traits were downloaded from GWASdb in July 2015 [23, 24], including all SNPs with nominal *P* < 1e-3 from the reported GWAS. Since GWASdb is a mixture of disease- and trait-associated SNPs from many different sources, we first picked the SNPs associated with a number of complex diseases including acute lung injury, Alzheimer’s disease, amyotrophic lateral sclerosis, asthma, bipolar disorder, cardiovascular disease, coronary heart disease, Crohn’s disease, major depressive disorder, multiple sclerosis, Parkinson’s disease, rheumatoid arthritis, schizophrenia, Type 1 diabetes, and Type 2 diabetes. For comparison, we then picked the SNPs associated with a number of phenotypic traits including blood pressure, body mass index, bone mineral density, cholesterol, fibrinogen, glucose, height, IgE levels, iron levels, lipid levels, lymphocyte counts, metabolite levels, odorant perception, red blood cell traits, taste, triglycerides, urate levels, waist circumference, and weight.

Genes implicated in autosomal recessive or autosomal dominant Mendelian disorders were compiled from a curated OMIM database available as supplementary datasets from a study by Petrovski et al. [33]. We used the original “OMIM recessive” genes as our recessive gene list, and we combined “OMIM dominant”, “OMIM de novo”, and “OMIM haploinsufficiency” genes together as our dominant gene list because for all of them one copy malfunction is sufficient to cause the disease.

### Estimating 10-year survival probabilities of biobank individuals

Based on EHR data of the Biobank individuals, we computed the Charlson probability [30, 31], an approximation of a patient’s 10-year survival probability. This measure is normally used to assess whether the patient will live long enough to benefit from a specific screening measure or medical intervention, and depends on patient’s age and clinical conditions they had in the past 5 years. Specifically, patients younger than 40 years old were given 0 point, patients between 41 and 50 years old were given 1 point, patients between 51 and 60 years old were given 2 points, patients between 61 and 70 years old were given 3 points, and patients between 71 and 80 years old were given 4 points. Clinical conditions were scored based on the risk of dying: myocardial Infarction (1 point), congestive heart failure (1 point), peripheral vascular disease (1 point), cerebrovascular disease (1 point), dementia (1 point), COPD (1 point), connective tissue disease (1 point), peptic ulcer disease (1 point), diabetes mellitus (1 point uncomplicated, 2 points if end-organ damage), moderate to severe chronic kidney disease (2 points), hemiplegia (2 points), leukemia (2 points), malignant lymphoma (2 points), solid tumor (2 points, 6 points if metastatic), liver disease (1 point mild, 3 points if moderate to severe), and AIDS (6 points). The Charlson probability was calculated as $Z={0.983}^{e^{\left(A+C\right)\ast 0.9}}$, where A is the age score and C is the summation of clinical condition scores. Since Charlson probability only applies to patients at or under 80, we removed 64 individuals above 80 years old. We also removed one individual with unknown gender, one individual without EHR, and one individual below 40 years old.

### Permutation test

To test if the heterozygosity rates between Wellderly and Biobank individuals are significantly different, the two cohorts’ genotype data were combined as an 852 by 100,206 matrix. For each SNP (column) in a permutation, we randomly picked 426 genotypes from the total 852 genotypes. We then combined the permuted columns to form 426 pseudo-individuals so that we could calculate heterozygosity rates for each pseudo-individual. We then calculated average heterozygosity rates for each round of permutation and compared with those from the Wellderly and Biobank cohorts. The permutation was run for 10,000 times in R [57].

### Significance test for the association between heterozygosity rate and 10-year survival probability

To test if the association between heterozygosity rate (HetRate) and 10-year survival probability (10ySP) was significant, we constructed a multiple linear regression model 10ySP ~ HetRate + age + comorbidity + gender + PC1 + PC2 + PC3 + PC4 + PC5, where comorbidity is the summation of clinical condition scores and PC1 to PC5 are the first five principal components from the PCA of the population structure. The modeling was implemented in R [57].

### Evolution and pathogenicity of the nonsynonymous SNPs

For the nonsynonymous SNPs, evolutionary probabilities were downloaded from myPEG (http://www.mypeg.info/home) [35], and Combined Annotation Dependent Depletion (CADD) scores were downloaded from dbNSFP [60, 61].

### Gene set enrichment analysis

Genes containing the top 10% excess of heterozygosity SNPs in each cohort (top genes) were used to test for gene set enrichment. We downloaded seven collections of gene sets from Molecular Signatures Database (MSigDB) v5.1: Hallmark, chemical and genetic perturbations, canonical pathways, GO biological process, GO cellular component, GO molecular function, and immunologic signatures [38]. For enrichment analysis, we performed hypergeometric test where the background is 4864 genes harboring the 7697 nonsynonymous SNPs and the tested gene sets are the intersection between the background and the gene sets from MSigDB collections. We corrected for multiple testing using Benjamini-Hochberg method of controlling the false discovery rate (FDR) in each of the gene set collections.

## Additional files

Additional file 1: **Figure S1.** Population structures of the Biobank and Wellderly individuals compared to the European populations of 1000 Genomes. A) PCA based on the SNPs with MAF > 0.2. B) PCA based on the SNPs with MAF > 0.01. (DOCX 219 kb)

Additional file 2: **Figure S2.** Population structures of the Biobank and Wellderly individuals before and after genetic matching. A) The original 1107 Biobank individuals and 454 Wellderly individuals. B) 454 matched pairs of Biobank individuals and Wellderly individuals. C) Distance for each one of the 454 matched pairs of Biobank-Wellderly individuals; the dashed horizontal line represents an arbitrary cutoff of distance 900. D) 426 matched pairs with distance less than 900. (DOCX 266 kb)

Additional file 3: **Figure S3.** Relationship between genetically matched Biobank cohort and Wellderly cohort on A) minor allele frequency (MAF), B) expected heterozygosity (HET_E_), C) observed heterozygosity (HET_O_), and D) excess of heterozygosity (F). (DOCX 340 kb)

Additional file 4: **Figure S4.** Heterozygosity comparison of noncoding SNPs between Biobank (orange) and Wellderly (green) after linkage disequilibrium based SNP pruning. A) Mean excess of heterozygosity. B) Number of SNPs showing higher ratio of Dd/DD in Biobank or Wellderly under different nominal *P* value cutoffs from Fisher’s Exact Test. C) Mean excess of heterozygosity for SNPs in different MAF bins; Numbers at the bottom of bars are SNP numbers in each bin. D) Mean excess of heterozygosity for SNPs associated with selected complex diseases (Diseases), selected phenotypic traits (Traits), and all the complex diseases and traits combined (All); Numbers at the bottom of bars are SNP numbers in each category. *P* values shown are raw values but with FDR < 0.05. (DOCX 122 kb)

## Data Availability

The Mount Sinai Bio*Me* Biobank data are available in dbGaP under accession number phs000925.v1.p1. The Wellderly cohort data were obtained by Material Transfer Agreement between Scripps Genomic Medicine and Icahn Institute for Genomics and Multiscale Biology.

## Abbreviations

EHR: Electronic health records
FDR: False discovery rate
FET: Fisher’s exact test
GWAS: Genome-wide association studies
HET_E_: Expected heterozygosity
HET_O_: Observed heterozygosity
HFC: Heterozygosity-fitness correlation
LD: Linkage disequilibrium
MAF: Minor allele frequencies
PCA: Principal component analysis
SNP: Single nucleotide polymorphism
