# Supplementary material for: Genome-wide analysis indicates association between heterozygote advantage and healthy aging in humans
Source: BMC Genet. 2019 Jul 2;20:52. doi: 10.1186/s12863-019-0758-4 (PMC6604157; doi:10.1186/s12863-019-0758-4)
Supplement: Supplementary file 1 — Figure S1. Population structures of the Biobank and Wellderly individuals compared to the European populations of 1000 Genomes. A) PCA based on the SNPs with MAF > 0.2. B) PCA based on the SNPs with MAF > 0.01. (DOCX 219 kb) [file 12863_2019_758_MOESM1_ESM.docx]

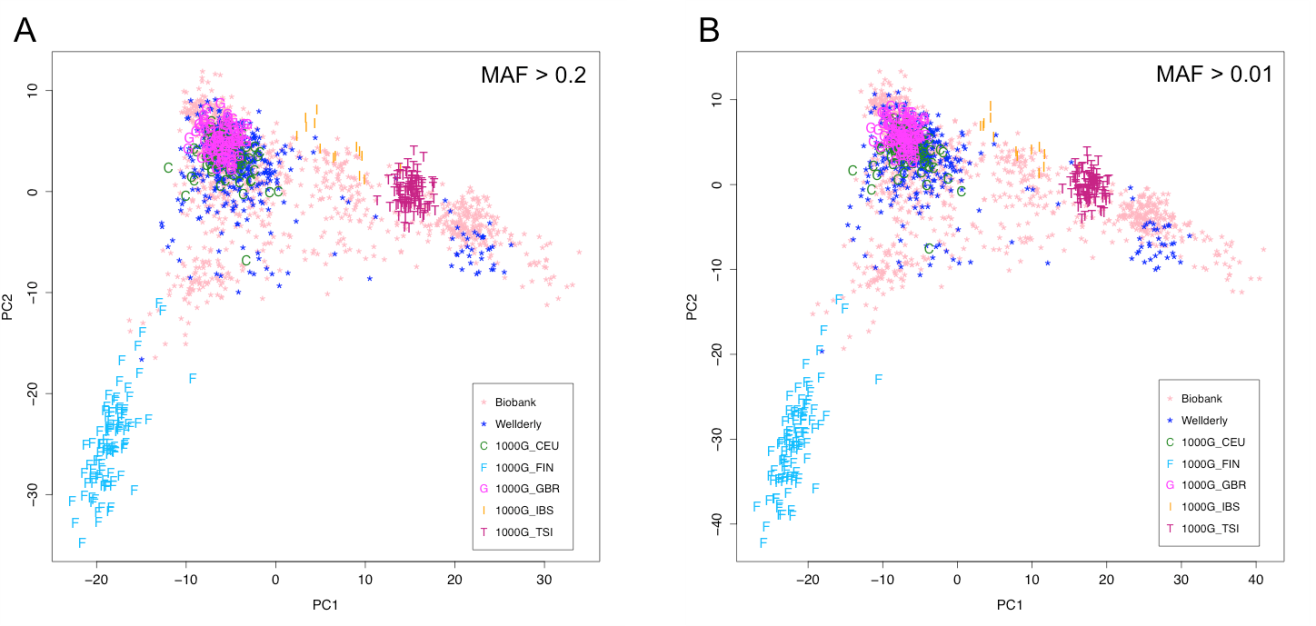


**Additional file 1: Figure S1.** Population structures of the Biobank and Wellderly individuals compared to the European populations of 1000 Genomes. A) PCA based on the SNPs with MAF > 0.2. B) PCA based on the SNPs with MAF > 0.01.
