# Supplementary material for: Genome-wide analysis indicates association between heterozygote advantage and healthy aging in humans
Source: BMC Genet. 2019 Jul 2;20:52. doi: 10.1186/s12863-019-0758-4 (PMC6604157; doi:10.1186/s12863-019-0758-4)
Supplement: Supplementary file 2 — Figure S2. Population structures of the Biobank and Wellderly individuals before and after genetic matching. A) The original 1107 Biobank individuals and 454 Wellderly individuals. B) 454 matched pairs of Biobank individuals and Wellderly individuals. C) Distance for each one of the 454 matched pairs of Biobank-Wellderly individuals; the dashed horizontal line represents an arbitrary cutoff of distance 900. D) 426 matched pairs with distance less than 900. (DOCX 266 kb) [file 12863_2019_758_MOESM2_ESM.docx]

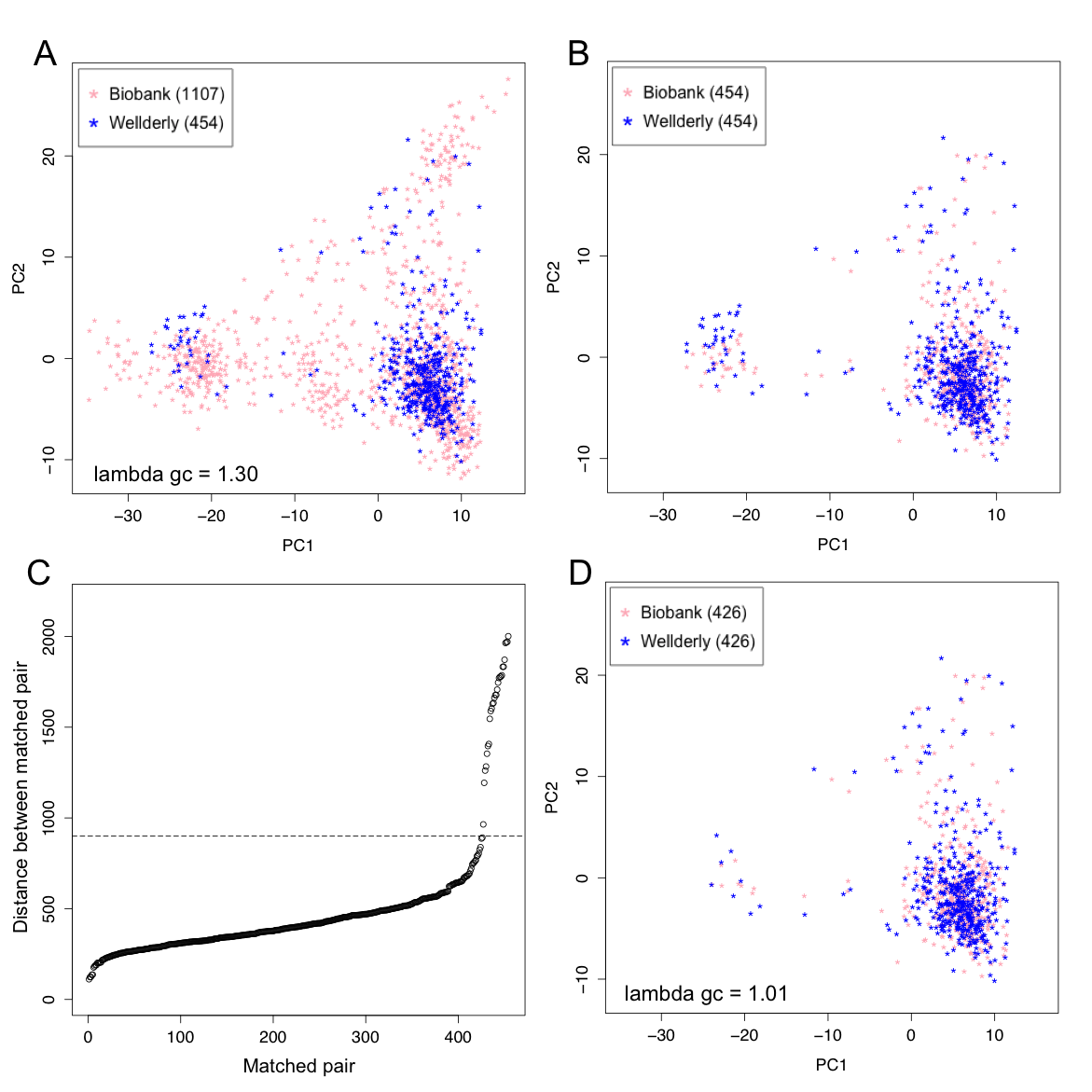


**Additional file 2: Figure S2.** Population structures of the Biobank and Wellderly individuals before and after genetic matching. A) The original 1,107 Biobank individuals and 454 Wellderly individuals. B) 454 matched pairs of Biobank individuals and Wellderly individuals. C) Distance for each one of the 454 matched pairs of Biobank-Wellderly individuals; the dashed horizontal line represents an arbitrary cutoff of distance 900. D) 426 matched pairs with distance less than 900.
