# Supplementary material for: Genome-wide analysis indicates association between heterozygote advantage and healthy aging in humans
Source: BMC Genet. 2019 Jul 2;20:52. doi: 10.1186/s12863-019-0758-4 (PMC6604157; doi:10.1186/s12863-019-0758-4)
Supplement: Supplementary file 3 — Figure S3. Relationship between genetically matched Biobank cohort and Wellderly cohort on A) minor allele frequency (MAF), B) expected heterozygosity (HETE), C) observed heterozygosity (HETO), and D) excess of heterozygosity (F). (DOCX 340 kb) [file 12863_2019_758_MOESM3_ESM.docx]

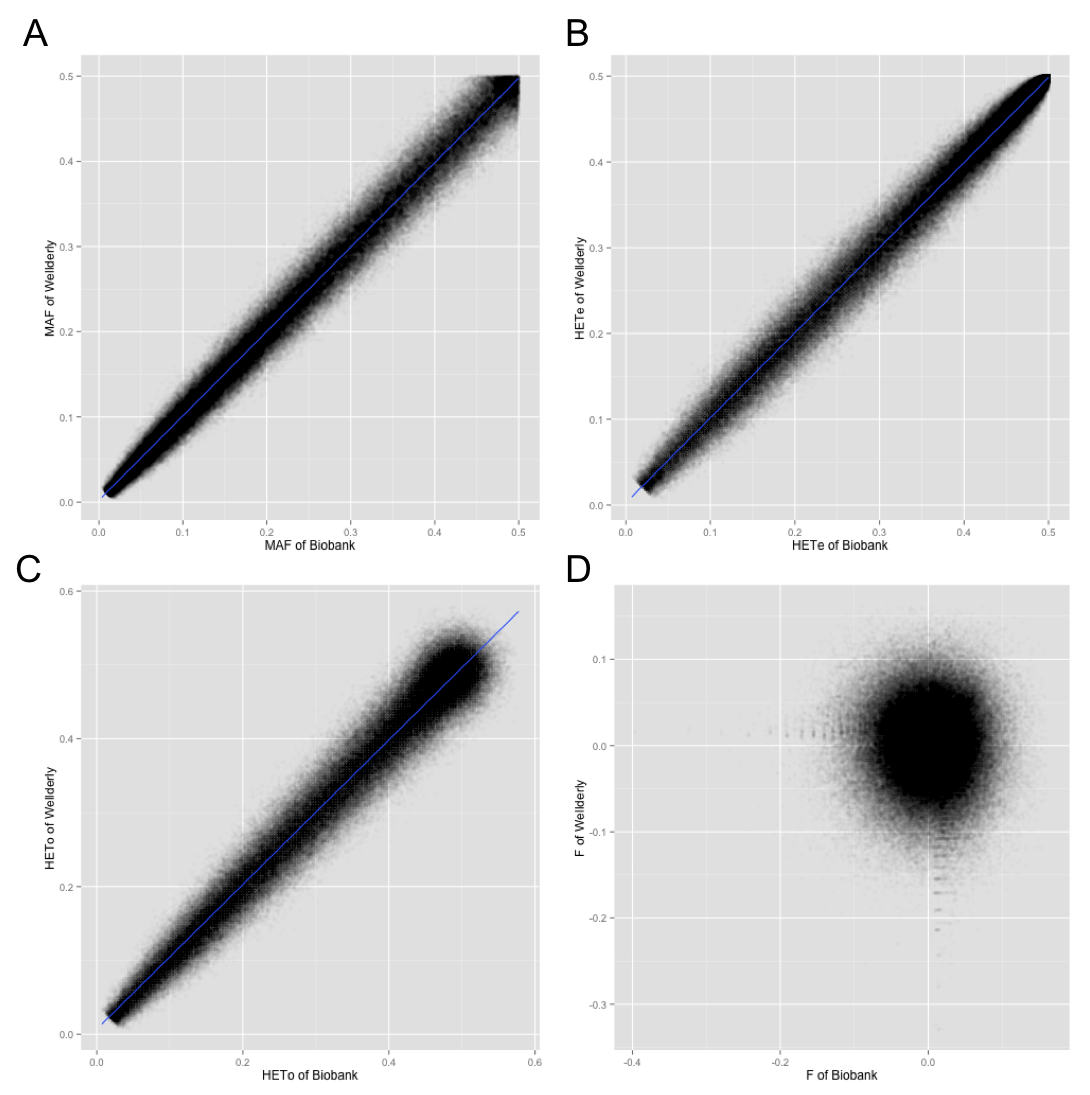


**Additional file 3: Figure S3.** Relationship between genetically matched Biobank cohort and Wellderly cohort on A) minor allele frequency (MAF), B) expected heterozygosity (HET_E_), C) observed heterozygosity (HET_O_), and D) excess of heterozygosity (F).
