# Supplementary material for: Genome-wide analysis indicates association between heterozygote advantage and healthy aging in humans
Source: BMC Genet. 2019 Jul 2;20:52. doi: 10.1186/s12863-019-0758-4 (PMC6604157; doi:10.1186/s12863-019-0758-4)
Supplement: Supplementary file 4 — Figure S4. Heterozygosity comparison of noncoding SNPs between Biobank (orange) and Wellderly (green) after linkage disequilibrium based SNP pruning. A) Mean excess of heterozygosity. B) Number of SNPs showing higher ratio of Dd/DD in Biobank or Wellderly under different nominal P value cutoffs from Fisher’s Exact Test. C) Mean excess of heterozygosity for SNPs in different MAF bins; Numbers at the bottom of bars are SNP numbers in each bin. D) Mean excess of heterozygosity for SNPs associated with selected complex diseases (Diseases), selected phenotypic traits (Traits), and all the complex diseases and traits combined (All); Numbers at the bottom of bars are SNP numbers in each category. P values shown are raw values but with FDR < 0.05. (DOCX 122 kb) [file 12863_2019_758_MOESM4_ESM.docx]

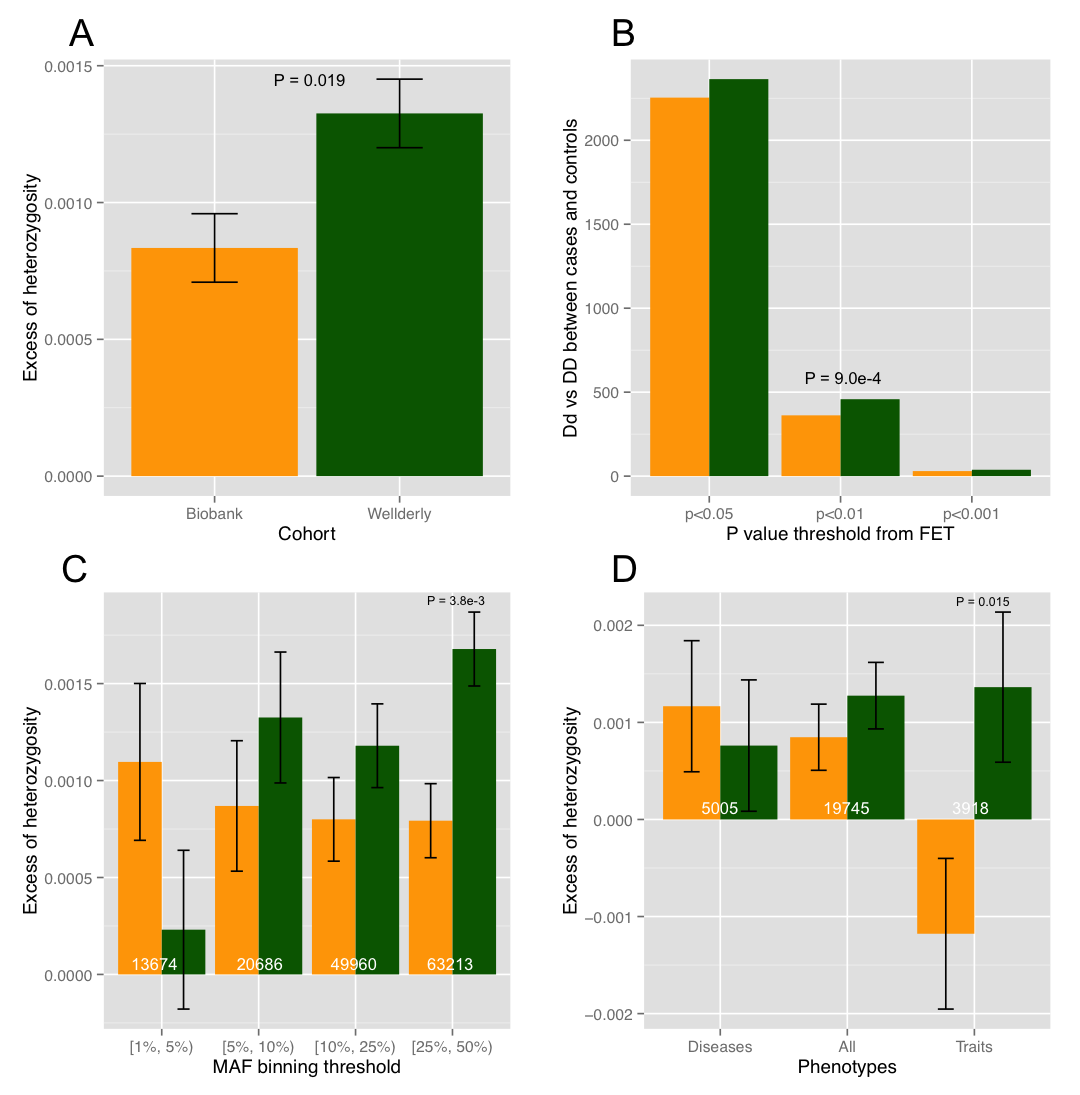


**Supplementary file 4: Figure S4.** Heterozygosity comparison of noncoding SNPs between Biobank (orange) and Wellderly (green) after linkage disequilibrium based SNP pruning. A) Mean excess of heterozygosity. B) Number of SNPs showing higher ratio of Dd/DD in Biobank or Wellderly under different nominal *P* value cutoffs from Fisher’s Exact Test. C) Mean excess of heterozygosity for SNPs in different MAF bins; Numbers at the bottom of bars are SNP numbers in each bin. D) Mean excess of heterozygosity for SNPs associated with selected complex diseases (Diseases), selected phenotypic traits (Traits), and all the complex diseases and traits combined (All); Numbers at the bottom of bars are SNP numbers in each category. *P* values shown are raw values but with FDR < 0.05.
